# Higher Dynamic Balance Performance Was Associated With Cognitive Function Among U.S. Community-Dwelling Low-Income Older Adults

**DOI:** 10.1177/23779608241296629

**Published:** 2024-11-13

**Authors:** Renata Komalasari, Elias Mpofu, Hui Chen (Rita) (Chang), Eva Berthy Talluntondok, Deby Kristiani Uligraff, Rongfang Zhan, Ladda Thiamwong

**Affiliations:** 1 Ross and Carol Nese College of Nursing, Pennsylvania State University, State College, PA, USA; 2 Keperawatan (Nursing), Tzu Chi Hospital Indonesia, Pantai Indah Kapuk, Jakarta, Indonesia; 3 Keperawatan (Nursing), Akademi Keperawatan Andalusia Jakarta, Jakarta, Indonesia; 4 College of Rehabilitation and Health Services, 3404 University of North Texas, Denton, USA; 5 Faculty of Health Sciences, University of Sydney, Camperdown, Australia; 6 School of Human and Community Development, University of Witwatersrand, Johannesburg, South Africa; 7 School of Nursing and Midwifery Faculty of Nursing, 6489 Western Sydney University, Penrith, Australia; 8 Faculty of Nursing, University of Pelita Harapan Nursing Department, Tangerang, Indonesia; 9 School of Health in Social Science, Nursing Studies, University of Edinburgh, Edinburgh, UK; 10 College of Nursing, 6243 University of Central Florida, Orlando, FL, USA; 11 Disability, Aging, and Technology Cluster, 6243 University of Central Florida, Orlando, FL, USA

**Keywords:** balance, cognitive function, gait, low-income older adults

## Abstract

**Introduction:**

Multiple studies have reported the association between balance performance and cognitive function among older adults. However, few studies have been conducted in low-income settings.

**Objectives:**

This cross-sectional study examined the relationships of dynamic and static balance with cognitive function in community-dwelling low-income older adults in Orlando, Florida.

**Methods:**

Dynamic balance was assessed with a Short Physical Performance Battery (SPPB) and static balance with BTracks Balance System. Rowland Universal Dementia Assessment Scale (RUDAS) assessed cognition and Patient Health Questionnaire-9 assessed depressive symptoms.

**Results:**

Multiple regression analysis showed dynamic balance was significantly associated with cognition, but static balance was not, controlling for depressive symptoms (*N* = 89, *M*_age_* *= 72.70, *SD* ± 6.95; females = 79). Specifically, higher dynamic balance was associated with better cognitive function. Cognitively healthy participants (RUDAS scores ≥23) scored 2.526 points higher in SPPB than those with possible cognitive impairment (RUDAS scores <23).

**Conclusions:**

Findings provide the evidence to suggest dynamic balance and cognitive function health disparities affecting older adults from low-income community. By implication, dynamic balance and cognitive function should be part of routine screening with older adults living in low-income communities.

## Introduction

Older adulthood is associated with various physical and cognitive challenges to community living and participation. From the aging processes, older adults may experience difficulties in maintaining balance associated with diminished cognitive performance (Li et al., 2019). For instance, neuromuscular function deteriorates with advancing age, contributing to muscle strength loss and poorer force control (Demnitz et al., 2016; Siriwardhana et al., 2018). Moreover, older adults are more vulnerable to a variety of neurodegenerative disorders conditions (e.g., Alzheimer's disease and related dementias) than the general population (Dumurgier & Tzourio, 2020), portending severe risk for loss of muscle strength and cognitive function. There is also evidence to suggest that older adults with higher activities of daily living limitations due to frailties and poorer cognition tend to come from lower socioeconomic backgrounds (Sánchez-García et al., 2014; Siriwardhana et al., 2018; Ton et al., 2017), implying a health disparity affecting that community population. Low socio-economic communities have ongoing health disparities from lower healthcare access and resources in old age (Komalasari et al., 2023b; Udoh et al., 2023). Moreover, higher frailty prevalence has been associated with women, who also carry a risk for neurodevelopmental conditions two times higher than for men (Dumurgier & Tzourio, 2020), of which the gendered health disparity effect would be larger in low socio-economic status communities. Those older adults of marginalized communities would bear the cumulative burden of lower access to healthcare resources (Sánchez-García et al., 2014; Siriwardhana et al., 2018; Ton et al., 2017), which may show variously by their gait capabilities and mental well-being indicators (Sánchez-García et al., 2014).

On the social gradient continuum, older adults of lower socioeconomic status are at high risk for aging process-related frailty and poorer cognitive function (Harber-Aschan et al., 2020) from poor nutrition, health care, and mental distress in managing their activities of daily living. Older adult populations have a high usage of outpatient services, nursing, and home care (Ton et al., 2017) and hospital in-patient and ambulatory costs (Leibson et al., 2015). Evidence suggests higher frailty and risk for faster cognitive decline among older adults of low socioeconomic status (Harber-Aschan et al., 2020; Maharani et al., 2023). Older adults’ higher risk for loss of gait is associated with lower engagement in instrumental activities of daily living or physical activities (Merom et al., 2012) which their partners or carers may do for them. However, the relative effect of the social gradient in gait and cognitive function among community-dwelling adults is less known, suggesting a need for study in that vulnerable population to inform interventions for their improved cognitive health and well-being (see Udoh et al., 2023).

## Review of Literature

### Association Between Balance and Cognitive Performance

Physical balance plays a crucial role in everyday living activities and comprises two types: static and dynamic balance performance, which are gait indicators vulnerable to impairment with aging (Casteran et al., 2016; da Silva Borges et al., 2014). Dynamic balance involves moving and activities such as walking while talking or changing direction (Divandari et al., 2023; Santamaría-Peláez et al., 2023). Static balance is more stationary, comprising simple actions like standing or maintaining an upright position (Divandari et al., 2023). Previous studies have demonstrated the link between mobility, physical function, and cognition (Demnitz et al., 2016; Clouston et al., 2013; Veronese et al., 2016; Yoon et al., 2018). Surprisingly, fewer studies have focused on the association between balance and cognition (Demnitz et al., 2016; Meunier et al., 2021) with inconsistent findings. In a systematic review of cross-sectional studies on the association between mobility and cognition performance among healthy older adults, of 642 studies reviewed, 25 examined the association between gait and cognition and only five studies had a focus on balance and cognition (Demnitz et al., 2016).

Most studies being reviewed showed significant association between executive function and processing speed with dynamic and static balance, with small to moderate effect sizes (Demnitz et al., 2016). However, there was no significant correlation of global cognition and episodic memory with balance (Demnitz et al., 2016). This lack of significance was also reported in a more recent systematic review and meta-analysis of 32 studies investigating the association between cognition and balance types among healthy older adults. The results showed a positive association between balance performance and cognitive function, with a weak correlation between dynamic balance and memory ability, but a strong correlation between balance and executive functioning (Divandari et al., 2023). Positive significant relationship between balance, as indicated by tandem test scores, and cognitive functioning were also found in two longitudinal studies, such that older adults with worse balance are more likely to have worse cognition (Meunier et al., 2021; Muhammad et al., 2023). It is possible that the relationship between balance and cognition may be domain specific (Divandari et al., 2023) and affected by other comorbid conditions, thus further investigation is needed. Another notable finding from this review of literature is that a substantial amount of research on frailty among older adults was conducted in higher-income country (Siriwardhana et al., 2018).

### Association Between Balance, Cognition, and Depressive Symptoms

Depressive symptoms have been associated with various poorer physical and cognitive outcomes, including impaired balance and gait, particularly among older adults (Chung et al., 2015; Snowden et al., 2015). Studies have consistently demonstrated that older adults with more depressive symptoms tend to exhibit worse balance and slower gait (Belvederi Murri et al., 2020). Conversely, decreased balance is also associated with an increased risk of depression among older adults. For example, a longitudinal study on the association between depression and cognition among older adults in Italia showed that those with lower balance (as assessed by the Short Physical Performance Battery [SPPB]) were more likely to experience depression four years later (Veronese et al., 2017).

This interplay between balance, cognition, and depressive symptoms is further illustrated in research showing that older adults with higher levels of depression tend to have greater postural sway, an indicator of poor balance, compared to those with lower levels of depression (Casteran et al., 2016). Moreover, depression has been shown to predict future cognitive disorders and accelerate the progression to conditions such as mild cognitive impairment (MCI) and dementia (Chung et al., 2015; Olaya et al., 2019). Given the bidirectional relationship between balance and cognitive decline, especially in low-income settings where access to healthcare and preventive measures may be limited, it is crucial to explore how these factors interact (Halahakoon et al., 2019). This research aims to clarify the specific types of balance impairments that are most strongly associated with cognitive decline among older adults, particularly in community-dwelling, low-income populations in the U.S., while accounting for the influence of depressive symptoms.

### Theoretical Foundation

Neurodegenerative cognitive aging theory proposes that age-related changes are triggered by irreversible physiological degeneration in the nervous system (McNeill, 2011; Wang & Petrella, 2019), contributing to gait and cognition issues. The most vulnerable areas affected by the aging process are the frontal lobes involved in balance and impulse control (Quadagno, 2017). There is evidence to suggest that the variance in cognitive function is associated with a sense of physical balance (Demnitz et al., 2016; Meunier et al., 2021). However, with advancing age, more cognitive functioning may be required to compensate for deficits in the physical balance-related sensorimotor system (Casteran et al., 2016). On the other hand, gait control or balance, a measure of mobility, may decline when there are deficits in motor and cognitive coordination (i.e., attention, executive functioning, and visuospatial processing (Casteran et al., 2016; Demnitz et al., 2016)). These may result in poorer health outcomes, such as limited quality of life and leisure activity engagement (Kim, 2016; Rivera-Torres et al., 2021) and higher risks of depression, which will eventually exacerbate cognitive health in later life (Casteran et al., 2016; Li et al., 2019). Therefore, this study aimed to cross-sectionally examine the association between dynamic, static balance performance, and cognitive performance measures.

The burden of care for older adults with neurodegenerative conditions is edging towards a crisis (Dorsey et al., 2013; Rapp et al., 2015) in the absence of evidence for informing preventive health care interventions among high-risk communities. Knowledge about the association between dynamic, static balance, and cognitive performance is important to inform interventions to improve gait and cognitive functioning among low-income older adults, thus enhancing their health-related quality of life.

## Methods

### Design

This cross-sectional study used baseline assessment data from a clustered randomized controlled trial study from January to December 2022.

**Variables and Measurement** Participants self-reported their socio-demographics (e.g., age, gender, education level, financial status, race/ethnicity group, health, and living arrangement). They responded to two types of balance performance measures (i.e., dynamic and static balance) and cognitive performance measure.

Dynamic balance performance was assessed by three physical function tests of the Short Physical Performance Battery (SPPB), a combination of gait speed, chair stand, and balance tests (Divandari et al., 2023). In the standing balance test, participants were asked to remain standing with their feet positioned together side by side, then in a semi-tandem stance (i.e., the heel of one foot placed to the first toe of the other foot). Each stance is required to be held for 10 s and if a participant fails one aspect of this test, the subsequent aspects will not be attempted. In the gait speed test, the time required to walk a 4-meter marked course at a usual pace was recorded. In the chair stand test, participants were asked to stand and sit in a chair five times as quickly as they could with arms crossed over the chest, and the time to complete five consecutive chair stands was recorded. For the standing balance test, the score was given based on the ability to maintain balance in each of the three stances. For the other two tests, scores are given, firstly, depending on the ability to complete the tasks and secondly, based on the time taken to complete each task. Each test is scored on a 0–4 scale. A total score for SPPB was obtained by summing up all three test scores and it ranged between 0 (*worst performance*) to 12 (*best performance*) (Divandari et al., 2023). SPPB has been used as a predictive tool for possible disability and can aid in the monitoring of lower limb function in older people. The reliability of SPPB scores in the previous study was good with* Cronbach α* of .86 (Santamaría-Peláez et al., 2023). The internal consistency of the items of the SPPB was acceptable (*Cronbach α *= .616).

Static balance performance was measured by the BTracks Balance System (BBS or BTrackS) scores. BBS is an effective, cost-effective, portable, and user-friendly assessment tool for assessing objective balance testing (Murray et al., 2018), particularly for older populations in clinics or communities (Thiamwong & Suwanno, 2014). This test consisted of four 20-s trials, and the score of each trial ranged from 0 to 100 and then summed up. A higher BBS score denotes poorer balance. In each attempt, the participants had to stand as still as possible on the BTrackS Balance plate with both hands on both sides of the hips and eyes closed. Scores from the BBS yielded excellent test-retest reliability over time in a previous study (Intraclass correlation = .685-.838) within 48–72-h intervals (Murray et al., 2018).

Cognitive performance was assessed by the Rowland Universal Dementia Assessment Scale (RUDAS) scores, with a value range of 0 to 30. The RUDAS is a six-item valid and reliable cognitive function assessment scale for older adults living in multicultural and language backgrounds (Komalasari et al., 2019; Storey et al., 2004). It assessed various cognitive domains: memory recall, body orientation, praxis (alternative hand movement), judgment, visuo-constructional (cube drawing), and semantic fluency (animal naming) (Nielsen & Jørgensen, 2020). For example, in memory recall items, participants were asked to recall a grocery list (tea, cooking oil, eggs, and soap) after five-minute intervals to assess memory capability. The RUDAS maximum score is 30; scores of < 23 signify possible cognitive impairment and scores ≥23 indicate normal cognition (Nielsen & Jørgensen, 2020). The RUDAS scale has 89% sensitivity and 98% specificity, with 99% inter-rater and 98% test-retest reliability (Nielsen & Jørgensen, 2020). The internal consistency of the items in this study was poor (*Cronbach's α *= .277).

Depressive symptoms were assessed by the nine items of the Patient Health Questionnaire-9 (PHQ-9) scale, a psychometrically sound nine-item tool to assess the severity of depression (Kroenke et al., 2001). The scale comprises statements such as “Feeling down, depressed or hopeless in the past two weeks” with a scale of 0–3. At a score of ≥10, PHQ-9 has a .88 sensitivity and .88 specificity for major depression (Kroenke et al., 2001). This scale has excellent internal consistency (*Cronbach α *= .89), as well as excellent sensitivity (.88 to .92) and specificity (.85 to .88) for use in the aging population (Kroenke et al., 2001; Levis et al., 2019); (Sun et al., 2020). The internal consistency of the items of the scale in this study is excellent (*Cronbach's α *= .854).

Covariate in this study is depressive symptoms. Previous studies have shown that depressive symptoms may co-occur with a cognitive decline among older adults (Casteran et al., 2016; Malatyali et al., 2023; Veronese et al., 2017)

### Research Questions

Following the neurodegenerative cognitive aging theory (McNeill, 2011; Wang & Petrella, 2019), this study aimed to answer the following research questions:

1. Would the dynamic balance performance be associated with cognitive performance among low-income older adults, such that higher dynamic balance would be associated with better cognition, controlling for depressive symptoms?
2. Would the static balance performance be associated with cognitive performance among low-income older adults, such that higher static balance would be associated with better cognitive function?

### Sample

Participants (*N *= 89; 79 females, *M*_age _= 72.70, *SD* ± 6.95) were recruited from low-income communities, units, apartments, and independent living communities in Central Florida. The average time to complete the full survey was approximately 30 min. The research coordinator checked all completed questionnaires to verify the quality of responses. Data were collected by a trained research assistant ensuring privacy, comfortable settings, and sufficient time for each participant when completing questionnaires.

### Inclusion and Exclusion Criteria

Participants were enrolled in the study if they met all of the following inclusion criteria: (1) adults aged 60+; (2) low-income (using poverty thresholds for 2019 by family size and number of children age <18, published by the U.S. Census Bureau), and (3) live in their own homes or apartments. Exclusion criteria: (1) a medical condition preventing balance test (e.g., inability to stand on the balance plate); (2) currently receiving rehabilitative treatment, and inability to speak English or Spanish.

### Institutional Review Board Approval

The study was approved by the institutional review board of the University of Central Florida. Informed consent was obtained and participants were compensated financially with gift cards. The Institutional Review Board at the University of Central Florida approved this study (see Thiamwong et al., 2020).

### Statistical Analysis

The sample size is estimated using G * power version 3.0.10 software and parameter settings (alpha = 0.05, power = 0.85, effect size f2 = 0.15). The estimated sample size was calculated, and a total of 76 participants plus 10%, equaling 83 participants, was considered sufficient (Faul et al., 2009). Statistical analysis was conducted using the Statistical Package for Social Sciences (SPSS) version 28 after data was cleaned and screened. One missing data in the age variable was treated with mean imputation. While the focus on this study is on the association between cognition and balance, two missing data on the RUDAS scores were treated with mean imputation given missing data is much less than 5% (Schafer, 1999). The mean and standard deviation were calculated for a normal distribution of continuous variables and percentages for categorical variables. A descriptive cross-sectional design was implemented to explore the correlation among the variables. After, the multiple regression analysis was employed to explore the association between sociodemographic factors with cognition, as measured by the RUDAS scores. Since the results were not significant, none of the sociodemographic factors was included in the regression model. Only depressive symptoms were significantly associated with the RUDAS scores, which were included as a covariate in the model.

All assumptions, such as multicollinearity, singularity, outliers, normality, linearity, and homoscedasticity, were ensured before running a standard multiple regression. Collinearity statistics were in the acceptable range: tolerance (range = .968 to .9879) and VIF (range = 1.013 to 1.033). Singularity of dynamic and static balance as independent variables was ensured as each is not a combination of others. The standardized residual plot shows that all values are between −3.0 and 3.0, indicating no outliers. As a follow-up, an independent *t*-test was run to examine the differences in dynamic balance performance across two groups: the cognitively healthy group (RUDAS scores ≥23) and possible cognitive impairment (RUDAS scores <23).

## Results

### Sample Characteristics

Table 1 presents demographic information for the sample and correlation among the variables. Participants were primarily female (88.8%), African American (71.9%), completed high school (57.3%), living alone (59.6%), just enough financially (56.2%) and had good health in general (57.3%). Most participants (87.60%) were in the cognitively healthy group (RUDAS scores ≥23) and 12.40% were in the possible cognitive impairment group (RUDAS scores <23). Based on the PHQ-9 scores, most participants (65.17%) had moderately severe to severe depressive symptoms (PHQ-9 score ≥10).

**Table 1.** Participant Characteristics.

Variables		Mean	*SD*
Age		72.70	6.95
		Frequency	Percentages (%)
Gender	Females	79	88.8
	Males	10	11.2
Education	Lower than high school	10	11.2
	High school	51	57.3
	College or above	28	31.5
Race/Ethnicity	African American	63	70.8
	Hispanic	21	23.6
	White	3	3.4
	Asian	2	2.2
Financial situation	Much less than adequate	6	6.7
	Less than adequate	18	20.2
	Just enough	50	56.2
	More than adequate	13	14.6
	Much more than adequate	2	2.2
Living arrangement	Living alone	53	59.6
	Living with family/friend	13	14.6
	Living with partner/spouse	21	23.6
Health status	Other	2	2.2
	Excellent	2	2.2
	Very good	20	22.5
	Good	52	58.4
	Fair	1	16.9

### Research Question Results

RQ 1. Would the dynamic balance performance be associated with cognitive performance among low-income older adults, such that a higher dynamic balance would be associated with better cognition, controlling for depressive symptoms?

Table 2 provides the Pearson correlations across all variables. Cognitive performance was positively associated with dynamic balance (*r* = .295, *p* = .005) and negatively associated with static balance (*r* = −.153, *p* = .153)*,* but not associated with depressive symptoms (*r* = −.017, *p* = .876). The multiple regression results indicated that the model explained 14.4% of the variance, *R*^2^=.100, *F*(3,88) = 3.14, *p* = .029. The dynamic balance (*b* = .326, *p* = .009) was significantly associated with cognitive function, controlling for depressive symptoms (see Table 3). Specifically, higher dynamic balance was associated with higher cognitive function regardless of depressive symptoms.

**Table 2.** Pearson Correlation Coefficients of Variables Among the Study Participants.

Variables	M ± SD	Skewness	Kurtosis	1	2	3	4
1. Cognitive performance	25.87 ± 3.05	–.479	–.141	1			
2. Dynamic balance performance	8.69 ± 2.60	–.732	–.218	.296**	1		
3. Static balance performance	36.95 ± 23.57	2.60	8.98	–.153*	–.147*	1	
4. Depressive symptoms	12.60 ± 4.85	1.86	3.21	–.017*	–.053*	–.093*	1

*Notes.* **p < .05; *p ≥ .05.

**Table 3.** Association Between Dynamic Balance, Static Balance, and Cognitive Performances Among Older Adults.

Variables	*b*	*β*	*T*	*p-value*
1. Dynamic balance performance	.326	.279	2.67	.**009**
2. Static balance performance	−.015	−.113	−1.08	.284
3. Depressive symptoms	−.008	−.012	−.119	.905

*Note:* Bold figure denotes significant result.

**Figure 1.**
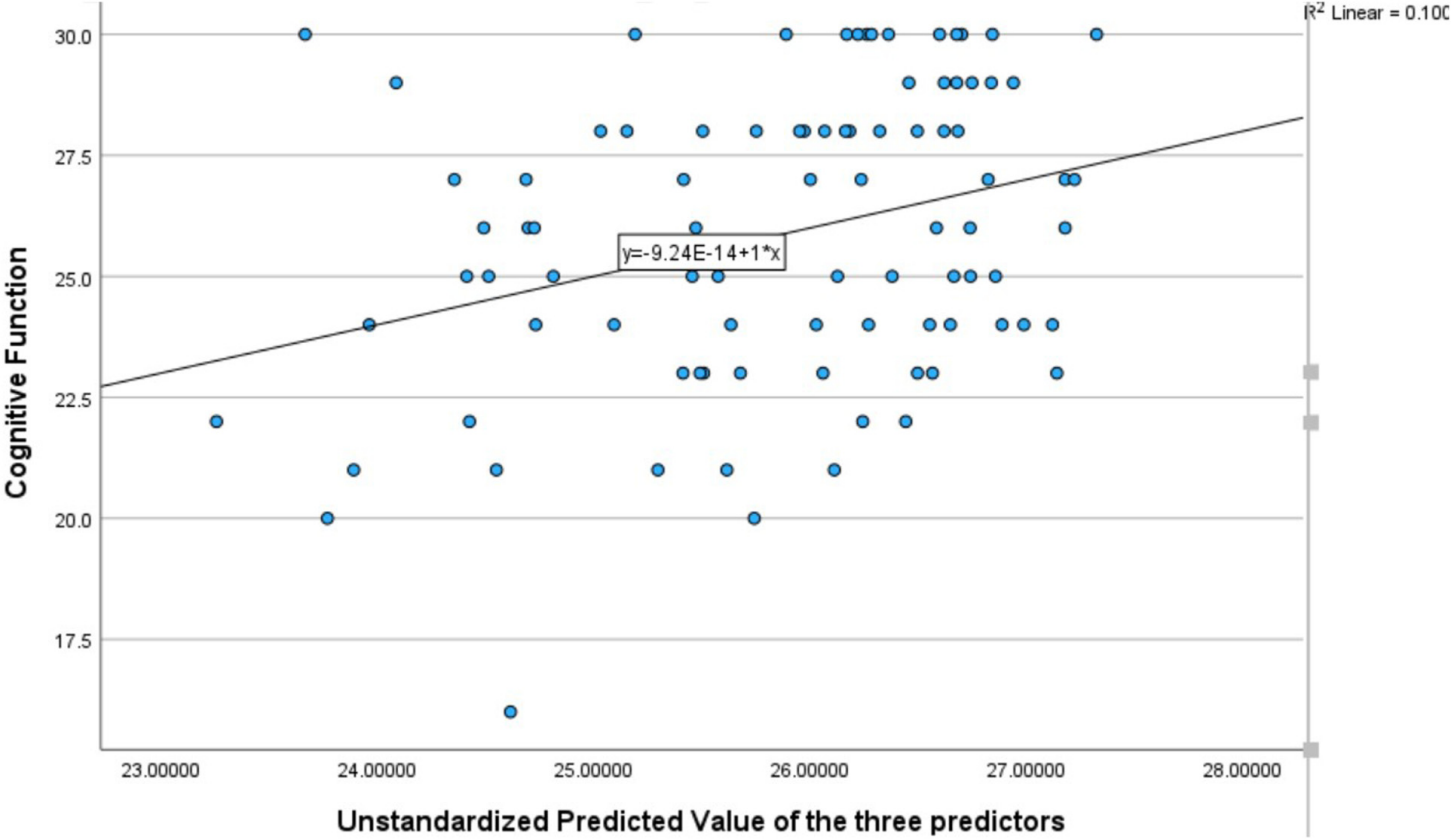
Scatterplot of the associations of dynamic balance and static balance with cognitive function, controlling for depressive symptoms.

Figure 1 shows scatterplots derived from the multiple regression results showing the association between unstandardized predicted values of dynamic balance and static balance and cognitive function, controlling for depressive symptoms.

Independent *t*-tests showed that there was a significant difference in the SPPB scores between the two groups of older adults, *F*(87) = .787, *p* = .001. Cognitively healthy older adults (mean = 9.026) scored 2.526 points higher in dynamic balance (SPPB) scores than those with possible cognitive impairment (mean = 6.50).

RQ 2. Would the static balance performance be associated with cognitive performance among low-income older adults, such that a higher static balance would be associated with better cognitive function?

Multiple regression analysis showed that static balance was not significantly associated with cognitive function (*b* = −.015, *p* = .284), controlling for covariate (*b* = −.008, *p* = .905).

## Discussion

Previous evidence is inconclusive on which types of balance performance (i.e., dynamic or static performance) are associated with cognitive performance. Additionally, a multitude of studies have examined older adults’ balance performance and cognitive performance, but few have been conducted among older adults in U.S. low-income settings. The study results showed that cognitive performance was associated with dynamic, rather than static balance, accounting for covariate. Specifically, older adults with higher dynamic balance performance tend to have higher cognitive functioning. Furthermore, cognitively healthy older adults performed better in dynamic balance than those with possible cognitive impairment. Static balance was not associated with cognition, controlling for covariate, which conflicts with the second hypothesis of this study.

The study findings and the possible mechanism underlying the association are consistent with the presupposition of cognitive aging theory (McNeill, 2011; Wang & Petrella, 2019). The biological aspect of this theory suggests that more cognitive functioning in older age may be required to compensate for deficits in the physical balance-related sensorimotor system (Tomaszewski Farias et al., 2018). As people get older, gait control or balance may decline due to deficits in motor and cognitive coordination (Demnitz et al., 2016; Morris et al., 2016), which may lead to poorer health outcomes, such as lower cognitive functioning (Demnitz et al., 2016; Li et al., 2019). These findings are also consistent with previous longitudinal evidence from a study among community-dwelling Italian older adults examining the associations of dynamic balance performance, semi-tandem, and handgrip strength with cognitive decline and impairment at 4.4 years follow-up, with dynamic balance performance being the strongest predictor of cognitive decline (Veronese et al., 2016). The findings of the current study build upon those of the study as this study also used the SPPB scores in assessing dynamic balance performance. However, in this study, the RUDAS scores were used to assess cognitive performance, which is a dementia assessment scale free from education bias or multicultural settings (Komalasari et al., 2023a; Nielsen & Jørgensen, 2020), suitable for use with older adults in low-income settings.

The findings of this study suggest no significant association between static balance and cognitive function in the older population, despite a significant correlation with dynamic balance. In contrast to previous research, Xiao and colleagues’ study found that cognitive function was related to both static and dynamic balance performance (Xiao et al., 2020). Moreover, the findings of the current study contrast with Leandri's cross-sectional study (2015) evaluating cognitive function and static balance with participants’ eyes closed. Their research, focusing on middle-aged and older adults, revealed a significant association between static and dynamic balance and cognitive function. These contrasting findings may arise from variations in study design, participant demographics, or measurement tools. Thus, a reevaluation of the cognitive demands associated with static balance tasks is warranted.

Additionally, the role of socioeconomic status (SES) must be considered when interpreting these findings. SES can influence access to healthcare, nutrition, physical activity, and other factors that contribute to both balance and cognitive function. The absence of an association between static balance and cognitive function might also reflect the limited cognitive demands posed by static balance tasks (Divandari et al., 2023), especially in populations facing socioeconomic challenges. Future research should further explore how SES interacts with balance and cognitive performance, and how these factors may differentially impact older adults in low-income settings.

## Strengths and Limitations

This study has the strength of providing evidence that lower dynamic balance may place older adults at higher vulnerability related to cognitive functioning, risking a lower quality of life (Kim, 2016; Li et al., 2019). Those lower on the socioeconomic gradient are particularly vulnerable and need routine screening services for their gait and cognitive performance for timely interventions. For study limitations, the researchers used a cross-sectional design, precluding causal explanations due to a lack of tracking of variable changes over time. Moreover, the study did not include measures of comorbid conditions as potential risk factors influencing balance and cognitive function among participants. The sample of older individuals was drawn from low-income communities in Orlando, Florida, and may not be representative of similar populations in other states or countries. For more dependable findings, a longitudinal design and a probability sample of low socioeconomic community adults should be considered in future research.

The demographic specificity of the study sample raises concerns about the generalizability of the study results, urging further studies that encompass more diverse populations to validate and extend these findings. Additionally, this study raises specific questions about the directionality of the relationship between dynamic balance and cognitive function. The identification of this association calls for cross-lagged design and/or longitudinal studies to elucidate whether improvements in dynamic balance can lead to enhanced cognitive function or vice versa. The findings would advance understanding of the dynamic interplay between balance and cognitive health in older adults for intervention design, implementation, and evaluation.

In this study, the dynamic balance was assessed by the Short Physical Performance Battery (SPPB), comprising gait speed, chair stand, and balance tests. Given previous evidence that the relationship between cognitive function and balance is task and cognitive domain-specific (Divandari et al., 2023), future research may investigate the association between each component of the SPPB separately to clarify the impact of the chair stand test on cognitive functioning.

## Implications for Practice

This study's findings have practice implications for targeted interventions and assessments tailored to the older adult population. To sustain cognitive capability, healthcare practitioners may consider incorporating dynamic balance tests as part of routine screenings as it may serve as a valuable tool for the early detection of cognitive impairment in older adults, enabling timely interventions. Older adults in deprived communities have less access to health care access and resources, risking avoidable deterioration in personal function. That being the case, it becomes critical for U.S. healthcare providers to optimize screening assessments for gait and cognitive performance for needed follow-up care using Medicare insurance provisions of which older adults with socioeconomic vulnerabilities may be less away (Farrell et al., 2022). Furthermore, incorporating dynamic balance exercises into fitness programs for older adults may be a practical and effective strategy for enhancing cognitive health. Cross-sectional study findings on frailty and cognitive performance among the aging population in low-income communities in the United States may be a basis for longitudinal studies to inform the design of interventions for successful aging in marginalized populations.

The researchers encourage healthcare practitioners and fitness professionals to consider these insights when developing exercise programs for older adults, especially those at higher risk of cognitive decline. Some strategies to improve balance in older people may include the tandem path, Swiss Ball exercises, physical exercise, and Hatha yoga (Sadondang & Komalasari, 2018) These insights into the interplay between dynamic balance and cognitive function provide actionable guidance for promoting overall health and functional independence in the older adult population.

## Conclusions

This study examined the association between dynamic, static balance, and cognitive performance measures in community-dwelling low-income older adults. This study indicated that dynamic balance was associated with cognitive performance, after adjusting for depressive symptoms. Incorporating a simple dynamic balance performance test which consists of standing balance, gait speed, and chair stand tests as part of routine screenings could serve as a valuable tool for early detection of cognitive impairment and to help monitor the cognitive changes in community-dwelling low-income older adults, enabling timely interventions. In addition, using the simple, reliable, and affordable strategy to assess dynamic balance may allow underserved older adults who have a high risk of cognitive decline to be reached on a larger scale. Findings highlight the need for targeting dynamic balance in both assessments and tailored interventions to maintain cognitive function in low-income older adults who have limited access to quality care.

## Supplemental Material

sj-docx-1-son-10.1177_23779608241296629 - Supplemental material for Higher Dynamic Balance Performance Was Associated With Cognitive Function Among U.S. Community-Dwelling Low-Income Older Adults Supplemental material, sj-docx-1-son-10.1177_23779608241296629 for Higher Dynamic Balance Performance Was Associated With Cognitive Function Among U.S. Community-Dwelling Low-Income Older Adults by Renata Komalasari, Elias Mpofu, Hui Chen (Rita) (Chang), Eva Berthy Talluntondok, Deby Kristiani Uligraff, Rongfang Zhan and Ladda Thiamwong in SAGE Open Nursing
